# Distribution of moniliformin in industrial maize milling and flaking process

**DOI:** 10.1007/s12550-024-00560-3

**Published:** 2024-09-11

**Authors:** Terenzio Bertuzzi, Alessio Abate, Paola Giorni

**Affiliations:** 1 Department of Animal Science, Food and Nutrition-DIANA, Università Cattolica del Sacro Cuore, Via Emilia Parmense 84, 29122 Piacenza, Italy; 2 Department of Sustainable Crop Production-DIPROVES, Università Cattolica del Sacro Cuore, Via Emilia Parmense 84, 29122 Piacenza, Italy

**Keywords:** Moniliformin, Maize, Milling process, Flaking process

## Abstract

Moniliformin (MON) is a widespread emerging mycotoxin often occurring in maize at significant levels. Few published studies investigated MON redistribution in maize-derived products for human consumption; to better understand this issue, 5 maize lots with different levels of MON contamination were processed following an industrial milling process to evaluate the redistribution of the mycotoxin in final products (grits), by-products destined to feed (bran and flour) and cleaning waste. MON was quantified by LC–MS/MS after the purification step through the SPE column; moreover, a confirmatory method based on MON derivatization with 1,2-diamino-4,5-dichlorobenzene was developed. Relevant MON reduction was obtained after sieve cleaning, scourer process, and optical sorting, achieving a decrement of the concentration level close to 70%. The following other milling procedures showed a limited reduction from cleaned maize to small and large grits; considering the entire industrial process, the reduction percentage of MON contamination in the final products was 80.9 ± 9.3% and 81.0 ± 6.7% for small and large grits, respectively. The flaking process showed a very limited reduction of MON, close to 10%. Considering the widespread of MON occurrence in maize, the study highlights the importance of cleaning steps to achieve a low risk of exposure for the consumer.

## Introduction

**Fig. 1:**
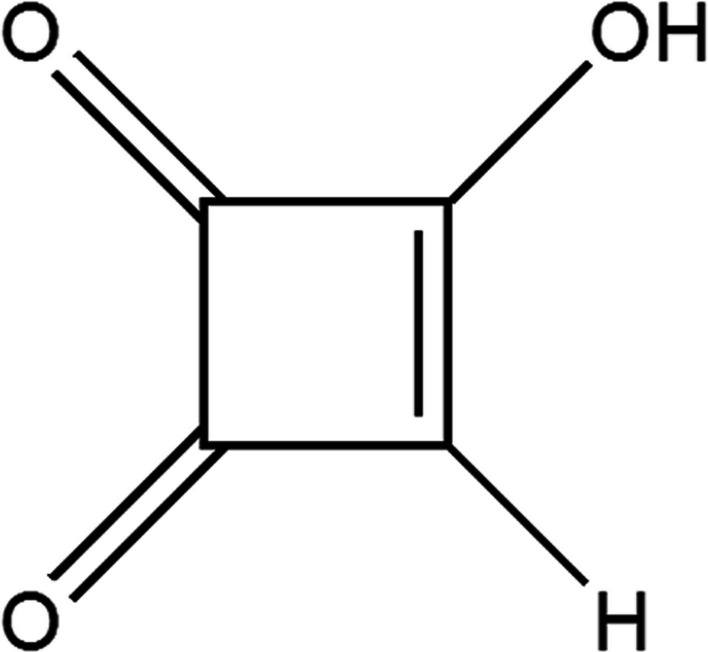
Structure of moniliformin

Moniliformin (MON) is an emerging *Fusarium* mycotoxin occurring in cereals with high levels found in maize. MON is mainly produced by *F. subglutinans*, *F. temperatum*, *F. verticilloides,* and *F. proliferatum* (Logrieco et al. 2002; Scauflaire et al. 2012; Scarpino et al. 2015); it is a highly polar and acidic molecule (Fig. 1) and occurs as a water-soluble sodium or potassium salt (Springer et al. 1974). The Panel on Contaminants in the Food Chain of the European Food Safety Authority (EFSA-CONTAM, 2018) indicated that cardiotoxicity and hepatotoxicity are its major adverse health effects, resulting in a shortage of energy, respiratory stress, and myocardial loss of functionality. MON can also increase oxidative stress, reducing the activity of glutathione peroxidase and glutathione reductase (Rossi, F.; et al. 2020); finally, its toxicity in combination with fumonisins was studied (Fremy et al 2019). No regulatory limits have been fixed by the EU Commission for MON; however, EFSA recommended more data on its occurrence in cereals and derived products. Widespread contaminations were found in cereals (mainly maize) cultivated in both Northern and Southern Europe, showing that MON can be produced in different climate conditions (Van Asselt et al. 2012; Scarpino et al. 2013; Herrera et al. 2017; Jajic et al. 2019; Bertuzzi et al. 2020; Janic et al. 2020). Although several studies on MON occurrence in maize have recently been published, few works investigated the distribution of this mycotoxin during the milling process. Pineda-Valdes reported a possible reduction of MON by heat processing and during alkaline cooking of maize (Pineda-Valdes et al. 2002, 2003; Tittlemier et al (2014) investigated the fate of MON during durum wheat milling, processing, and cooking of spaghetti. Lately, Scarpino et al (2020) determined a relevant reduction of MON during the maize large-scale dry milling process. In this work, the distribution of MON during the industrial dry milling process of different lots of maize intended for human consumption was evaluated. Five maize lots having different MON contaminations were processed and cleaning waste, by-products, and final products were collected and analyzed; finally, the influence of the industrial flaking process for corn flakes production on MON contamination was determined.

## Materials and methods

### Maize milling process and sampling

Five different commercial lots of maize cultivated in 2023 in Northern Italy were processed in an industrial mill. Initially, maize was subjected to different cleaning steps; at first, a sieve cleaner was used to discard broken grains having a particle size inferior to 500 µm. Then, maize was passed to densimetric tables to separate defective grains and successively to a destoner to remove impurities. After a new passage to densimetric tables, maize was subjected to an intensive scourer with an aspirator. A final cleaning step involved optical sorting to remove grains undetectable by visual control. Cleaned maize was processed using a tempering degermination system consisting of adding warm water to achieve a moisture level of about 20%; in these conditions, the germ becomes elastic and maize can be degerminated. After that, maize was sifted to obtain bran, flour, small, and large-size grits. Flour, bran, and a fraction of cleaning waste were processed for the production of animal feed flour. The sampled products represented a maize lot of about 40 tons and were collected during the milling process according to European Commission Regulation EC 401/2006 (European Commission 2006). From the 5 maize lots considered, unprocessed maize, two cleaning waste (after sieve cleaner and after densimetric tables; it was not possible to collect scourer waste), maize before and after optical sorting, germ, bran, flour, small and large size grits were collected. For each product, the final sample (about 5 kg) was obtained by blending 30 incremental samples (150–200 g each) collected at regular intervals for 1 h by means dynamic sampling procedure. The samples were kept at − 20 °C until the analysis. Moreover, two lots of cleaned maize were collected before and after an industrial flaking process. After a heating step close to 60 °C for 15 min and water addition, boiling was carried out at 120 °C and 150 kPa for 35 min before the flaking process. Then, a tempering step at 68 °C for 2 h and 30 min was carried out.

### Reagents and standards

The chemicals and solvents used for the extraction and clean-up steps were ACS grade or equivalent (Carlo Erba, Milan, Italy). For LC–MS/MS analysis, water, methanol, acetonitrile ammonium formate, and formic acid were LC–MS grade (Merck, Darmstadt, Germany). MON and 1,2-diamino-4,5-dichlorobenzene (DDB) were purchased from Sigma (St. Louis, MO, USA). For MON, a stock solution of 10 mg l^−1^ was prepared in water-acetonitrile 1 + 9 v/v; working solutions of 10–2000 µg l^−1^ were prepared by diluting with water-acetonitrile 1 + 9 v/v.

### LC–MS/MS analysis for MON determination

After milling of samples using a cyclone hammer mill (sieve 1 mm) and their homogenization, MON was extracted from 25 g using 100 ml mixture CH_3_CN:H_2_O 1 + 1 (v/v) for 60 min. After filtration on folded filter paper and centrifugation (3000 × *g* for 10 min), the extract was purified on a MycoSep® 240 Mon cleanup columns (Romer Labs®, Tulln, Austria); then, after filtration through 0.45 µm filter, the extract was injected (20 µl) into the LC–MS/MS system. The HPLC–MS/MS system consisted of an LC 1.4 Surveyor pump (Thermo Fisher Scientific, San Jose, CA, USA), a PAL 1.3.1 sampling system (CTC Analytics AG, Zwingen, Switzerland), and a Quantum Discovery Max triple quadrupole mass spectrometer; the system was controlled by an Excalibur 1.4 software (Thermo Fisher Scientific, San Jose, CA, USA). MON was separated on a HILIC zwitterionic column (BEH-Z-HILIC, 2.5 µm, 2.1 × 100 mm, Waters) using as mobile phase a mixture of 25 mM ammonium formate (pH 3.2):CH_3_CN 15 + 85 (v/v). The ionization was carried out with an ESI interface (Thermo-Fisher) in negative mode as reported by Bertuzzi et al. (2020): spray capillary voltage was 3.5 kV, sheath and auxiliary gas 40 and 15 psi, respectively; skimmer 9 V, the temperature of the heated capillary 350 °C. The mass spectrometric analysis was performed in selected reaction monitoring (SRM). For fragmentation of the [M − H]^−^ ion (97 m/z), the argon collision pressure was set to 1.2 mTorr and the collision energy to 21 V. The detected and quantified fragment ion was 41 m/z. Three replicates were performed for each sample.

The accuracy of the method was evaluated by spiking samples of maize flour, bran, germ, and siever cleaner waste with an appropriate volume (between 50 and 200 μl) of MON standard solution at three levels (100, 250, and 500 µg kg^−1^); three replicates were carried out for each level. As regards the matrix effect, defined as the difference between the mass spectrometric response for the analyte in standard solution and the response for the same analyte at the same concentration in matrix extract, a MON standard solution (100 µg l^−1^) in CH_3_CN:H_2_O 15 + 85 and spiked extracts at the same concentration of maize flour, germ and bran not naturally contaminated with MON were compared. For siever cleaner waste, the method of standard addition calibration was applied, adding increased amounts of analyte standard (three fortifications at 1000, 1500, and 2000 µg l^−1^) to several sample aliquots of a naturally contaminated sample, according to Cuadros-Rodriguez et al. (2007). The matrix effect was below 6% for maize flour, germ, and bran and 15% as a reduction for siever cleaner waste.

After evaluation of the matrix effect, the limit of detection (LOD) and quantification (LOQ) were determined. The LOD was defined as the level corresponding to a signal-to-noise ratio (S/N) of three, and the LOQ was the lowest level for which the repeatability of the analysis was below 10%, as described by Hartmann et al. (1998). The limit of detection (LOD) and quantification (LOQ) were 10 and 30 µg kg^−1^, respectively, for maize flour, germ, and bran, while for siever cleaner waste were 25 and 60 µg kg^−1^, respectively.

Satisfactory linearity (*r* > 0.998) was evaluated by injection of five MON standard solutions (5, 25, 100, 200, and 400 µg l^−1^). Each standard was injected twice; for the calibration curve, the average of two standards at the same concentration was considered.

### Confirmatory LC–MS/MS method for MON determination

Considering the low molecular mass of MON and the presence of only one transition, due to the production of one fragment ion using the conventional LC–MS/MS method for MON determination, derivatization with 1,2-diamino-4,5-dichlorobenzene (DDB) was carried out to increase the sensitivity and the accuracy of the analysis, in according to the study of Zollner et al. (2003). A total of 20 samples were analyzed using this method. Then, 2 ml of standard MON solutions and sample purified extracts (2 for each matrix) were derivatized with 0.5 ml DDB solution (1 mg/ml in HCl 1 M) at 60 °C for 120 min. After evaporation under a gentle flow of nitrogen, the extract was redissolved with 0.5 ml CH_3_CN:H_2_O 3 + 7 (v/v) and injected into the LC–MS/MS system. The HPLC–MS/MS system consisted of a Vanquish pump and autosampler, and a Fortis triple quadrupole mass spectrometer (Thermo Fisher Scientific, San Jose, CA, USA); the system was controlled by an Excalibur 1.4 software (Thermo Fisher Scientific, San Jose, CA, USA). MON was separated on a Betasil RP-18 column (2.5 µm, 2.1 × 100 mm, Thermo Fisher) using a gradient elution H_2_O (A):CH_3_CN (B), both acidified with 0.2% formic acid, as follows: a from 70 to 15% from 0 to 5 min, then isocratic for following 4 min and conditioning at initial percentages for 7 min. The ionization was carried out in positive mode with an H-ESI interface: sheath and auxiliary gas 35 and 12 psi, respectively; sweep gas 2 psi, temperature of heated capillary and vaporization 270 and 200 °C, respectively. The mass spectrometric analysis was performed in selected reaction monitoring (SRM). For fragmentation of the [M + H]^+^ ion (229 m/z), the dwell time was 500 ms, the argon collision pressure was set to 1.5 mTorr and the collision energy to 30 V. The fragment ions were 124, 152, and 187 m/z. The retention time of MON was 8.4 min (Fig. 1); the peak at 7.18 min was probably an isomer of MON derivative, already reported by Zollner et al. The identification of MON was based on the retention time and the presence of coinciding peaks for the selective transitions in the correct abundance ratio. The ratio of the product ions (least abundant/most abundant) in the samples was consistent with that obtained in the standard solution and, in any case, did not deviate more than 20%. The matrix effect was inferior to 5%; the limit of detection (LOD) and quantification (LOQ) were lower than the previous method, of 0.5 and 2 µg kg^−1^, respectively.

## Results and discussion

**Fig. 2:**
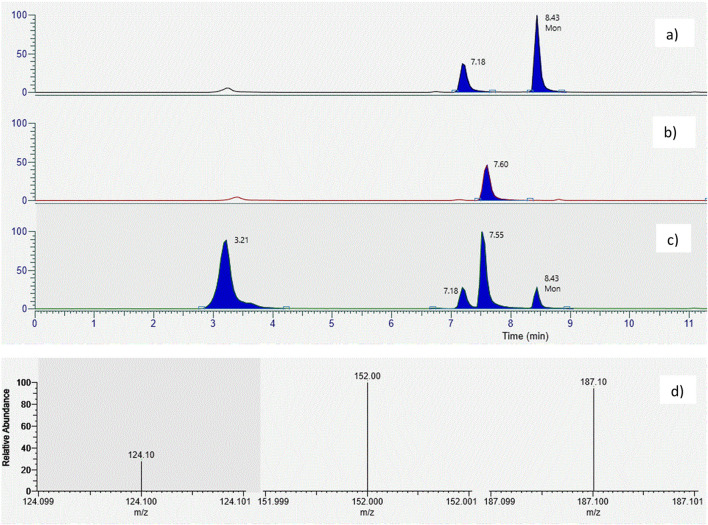
Chromatogram using the confirmatory method of **a** derivatized Moniliformin standard (1 mg/l; retention time 8.43 min); **b** derivatized blank solution; **c** derivatized cleaning waste extract of milling process; **d** selected fragment ions of derivatized moniliformin

The unprocessed maize lots showed different levels of MON contamination, from about 150 to 1100 µg kg^−1^, close to findings of previous surveys on maize cultivated in Northern Italy (Scarpino et al. 2015; Bertuzzi et al. 2020). Table 1 shows average contamination levels of MON (µg kg^−1^) found in the collected samples and for each lot, the ratio of distribution, calculated as MON concentration in the product/MON concentration in unprocessed maize. For each matrix, the standard deviations among the replicates were satisfactory and below 11.4%. The samples (*n* = 20) analyzed using the confirmatory LC–MS/MS method showed levels of contamination similar (± 7%) to those obtained with the conventional method (Fig. 2).

**Table 1:** MON contamination (µg/kg) in maize fractions collected during the milling process

	Maize 1	Ratio	Maize 2	Ratio	Maize 3	Ratio	Maize 4	Ratio	Maize 5	Ratio
Unprocessed maize	574.3		482.1		153.2		1090		755.4	
Waste of sieve cleaner	5099	8.88	2059	4.27	645.9	4.22	9167	8.78	3602	4.90
Maize with particle size > 500 µm	325.5	0.61	420.7	0.87	118.5	0.77	421.5	0.39	524.4	0.69
Waste of densimetric tables	475.5	0.83	203.4	0.42	515.6	3.37	188.1	0.17	185.8	0.25
Cleaned maize after optical sorting	131.8	0.23	119.6	0.25	59.5	0.39	256.8	0.24	253.0	0.33
Small grits	32.3	0.006	92.5	0.019	33.2	0.22	192.4	0.18	237.9	0.31
Large grits	53.7	0.009	82.9	0.027	37.8	0.25	195.5	0.18	195.8	0.26
Germ	21.4	0.004	40.0	0.008	26.1	0.017	60.1	0.006	160.9	0.21
Bran	930.2	1.62	304.5	0.63	177.8	1.16	1205	1.11	1119	1.48
Flour	263.6	0.46	107.7	0.22	91.7	0.60	291.4	0.27	343.2	0.45

The results confirmed the efficacy of cleaning steps before the milling process, mainly the use of sieve cleaner that removed high levels of MON with an average concentration in the waste about 6 times higher than in unprocessed maize (6.21 ± 2.40); the concentration in maize after this cleaning step was one third lower than in raw maize (mean 0.67 ± 0.18). The waste of densimetric tables resulted in less contamination, at a concentration close to that found in the initial maize (mean 1.01 ± 1.34). After the scourer process and the optical sorting, MON concentration in cleaned maize was about one-third of that in the unprocessed maize (average ratio 0.29 ± 0.07), achieving a global reduction of 71.3 ± 7.1%, remarkably higher than the values between 36–59% reported by Scarpino et al. (2020). The use of the optical sorter was confirmed to achieve a high reduction of MON contamination.

The following steps of the milling process showed high levels of contamination in bran fraction, reporting concentrations always higher in comparison with cleaned maize (average ratio 3.93 ± 1.97). Germ was the fraction less contaminated, and the average concentration ratio between germ and cleaned maize was 0.36 ± 0.19; higher ratios, but always inferior to 1, were calculated for both small and large size grits, showing very similar values, 0.65 ± 0.27 and 0.65 ± 0.15, respectively. Considering the entire process, MON concentration in both grits was one fifth than that found in unprocessed maize (0.19 ± 0.06 and 0.19 ± 0.09); then, the reduction percentage in the final product with respect to the unprocessed maize was 80.9 ± 9.3% and 81. ± 6.7% for small and large grits, respectively, resulting very similar to 80% and 64% reported by Scarpino et al (2020) for flaking and medium hominy grits. However, in our study, the concentration of MON in large and small grits was very similar.

Considering all five lots of maize, the average yield of the cleaning fractions was 3% for both sieve cleaner and densimetric table waste; generally, cleaned maize after optical sorting was 92% of the unprocessed maize. For the degermination and milling process, the average yields with respect to the cleaned maize were 2% for bran, 5% for germ, 20% for flour, and 55 and 10% for large and small-size grits, respectively. Considering these yields, Table 2 shows the amount (µg) of MON found in 100 kg of unprocessed maize and in the collected fractions.

**Table 2:** MON presence (µg) in maize fractions collected during the milling process

	Maize 1	Maize 2	Maize 3	Maize 4	Maize 5
Unprocessed maize	57,425	48,216	15,324	108,979	75,547
Waste of sieve cleaner	18,782	6176	1938	46,983	13,636
Maize with particle size > 500 µm	34,189	40,812	11,500	40,466	50,871
Waste of densimetric tables	1427	610	1547	376	558
Cleaned maize after optical sorting	12,127	11,004	5483	23,622	23,275
Small grits	323	926	332	1924	2380
Large grits	2955	4563	2081	10,756	10,772
Germ	107	200	131	301	805
Bran	1860	609	356	2410	1206
Flour	5271	2155	1835	5828	6865

The percentage of MON content found in siever cleaner and densimetric tables with respect to unprocessed maize were different among the lots; the average values, with high standard deviations, were 18.3 ± 7.0% and 3.0 ± 4.0%, respectively. However, after the entire cleaning process, the average percentage of MON that remained in the cleaned maize was 26.4 ± 6.5%, much lower with respect to the difference between MON in unprocessed maize and MON in siever and densimetric waste. These data showed that, besides siever cleaner and densimetric table waste, also the intensive scourer and optical sorting steps were very effective in removing the mycotoxin. Regarding degerminating and milling process, the sum of MON in bran, germ, flour, and grits achieved 86.9 ± 6.5% with respect to the MON in cleaned maize; this minor content found in the fractions can be due to the complexity of the sampling of several matrices. If compared to MON content in cleaned maize, MON distribution was 39.1 ± 8.9% in large-size grits, 7.1 ± 2.9% in small-size grits, 30.1 ± 9.1% in flour, 8.5 ± 4.3% in bran, 2.0 ± 1.0% in germ. These results showed that, after the milling process, a relevant amount of MON, about 50%, remained in final products (grits) respect the content in cleaned maize, showing a minor efficacy to reduce MON respect to the cleaning steps.

Finally, the flaking process showed a very low reduction of MON (mean of 2 processes was 10.1%), showing that this mycotoxin is stable at temperatures close to 100 °C. The contamination levels of MON before and after are reported in Table 3.

**Table 3:** MON contamination (µg/kg) before and after the flaking process

	Maize grits	Maize flakes	Reduction (%)
Lot 1	169.1	149.2	11.7%
Lot 2	41.8	38.2	8.6%

These results were similar to the findings of Pineda-Valdes et al. (2003), which observed a slightly higher reduction of MON, close to 20%, in corn grits extruded at temperatures between 140 and 180 °C.

## Conclusions

It is well known that fumonisins (FBs) and MON often co-occur in maize; FBs redistribution was largely investigated in the milling fractions showing high reduction both in cleaning and in the milling process; unlike MON was rarely studied. This study underlines that the cleaning steps are very efficient in reducing the content of MON in maize, as for FBs, and these operations are essential to reduce the risk of contamination of these *Fusarium* toxins. Moreover, the moderate reduction of MON during the following steps of milling might lead to a not negligible risk of exposure for the consumers, if a not accurate cleaning was carried out. The co-occurrence of MON and FBs was widely studied in maize samples; however, there are few data regarding maize-derived products intended for human consumption. The exposure to these mycotoxins could be relevant for consumers in countries where maize is a staple food, for the baby food supply chain, and for the celiac population. The confirmatory method for MON determination developed in this study was achieved to quantify MON at low levels confirming its identification through the correct abundance ratio of fragment ions.

## Data Availability

No datasets were generated or analysed during the current study.
