# Correction: Distribution of moniliformin in industrial maize milling and flaking process

**DOI:** 10.1007/s12550-025-00610-4

**Published:** 2025-09-06

**Authors:** Terenzio Bertuzzi, Alessio Abate, Paola Giorni

**Affiliations:** 1 Department of Animal Science, Food and Nutrition-DIANA, Università Cattolica del Sacro Cuore, Via Emilia Parmense 84, 29122 Piacenza, Italy; 2 Department of Sustainable Crop Production-DIPROVES, Università Cattolica del Sacro Cuore, Via Emilia Parmense 84, 29122 Piacenza, Italy

**Correction: Mycotoxin Research (2024) 40:659–665**

10.1007/s12550-024-00560-3

In the original version of this article, the given and family names of "Terenzio Bertuzzi, Alessio Abate, Paola Giorni" were incorrectly structured. Given here are the corrected author names.

The original article has been corrected.

